# New technologies in oral radiology as a diagnostic aid for monostotic fibrous dysplasia: a review

**DOI:** 10.21142/2523-2754-0904-2021-089

**Published:** 2021-12-09

**Authors:** Ximena Torrico-Acha, Jhoana Mercedes Llaguno-Rubio

**Affiliations:** 1 Facultad de Odontología, Universidad Mayor de San Simón. Cochabamba, Bolivia. xitocha_17@hotmail.com Universidad Mayor de San Simón Facultad de Odontología Universidad Mayor de San Simón Cochabamba Bolivia xitocha_17@hotmail.com; 2 División de Radiología Bucal y Maxilofacial de la Universidad Científica del Sur. Lima, Perú. academico@ilaeperu.com Universidad Científica del Sur División de Radiología Bucal y Maxilofacial Universidad Científica del Sur Lima Peru academico@ilaeperu.com

**Keywords:** Monostotic fibrous dysplasia, conventional radiography, digital radiography, cone beam computed tomography, magnetic resonance imaging, bone scintigraphy, displasia fibrosa monostótica, radiografía convencional, radiografía digital, tomografía computarizada de haz cónico, resonancia magnética, gammagrafía ósea

## Abstract

Monostotic fibrous dysplasia is a benign asymptomatic lesion that affects only one bone, which is replaced by amorphous connective tissue. Clinically there is an increase in the volume of the affected area, which is observed by imaging as a radiopaque area with diffuse non-corticalized limits capable of expanding to neighboring structures, and it is histologically evidenced as “resembling Chinese characters”. The lesion is seen as a radiopaque image with diffuse borders in conventional or digital radiography, while cone beam computed tomography identifies the exact location and extension of an isodense, mixed or hyperdense image of non-corticalized edges. Magnetic resonance imaging is also used when the lesion involves soft tissues or nerves, and bone scintigraphy is performed in order to systemically observe bone quality. The objective of this article was to describe the new technologies in oral radiology for the diagnosis of monostotic fibrous dysplasia and the importance of the current imaging methods in achieving an adequate diagnosis. These techniques range from conventional radiography to bone scans, which provide images of higher quality, clarity and better precision with less invasive techniques to the patient. This review of the literature helps to expand the knowledge of dental professionals in relation to the clinical and imaging characteristics of monostotic fibrous dysplasia.

## RESUMEN

La displasia fibrosa monostótica es una lesión benigna y asintomática que afecta solo a un hueso, que es reemplazado por tejido conectivo amorfo. Clínicamente, existe un aumento del volumen de la zona afectada, que se observa en la imagen como un área radiopaca con límites difusos no corticalizados capaces de expandirse a estructuras vecinas y se evidencia histológicamente como “semejanza de caracteres chinos”. La lesión se ve como una imagen radiopaca con bordes difusos en una radiografía convencional o digital, mientras que la tomografía computarizada de haz cónico identifica la ubicación exacta y la extensión de una imagen isodensa, mixta o hiperdensa de bordes no corticalizados. La resonancia magnética también se usa cuando la lesión involucra tejidos blandos o nervios, y se realiza una gammagrafía ósea para observar sistémicamente la calidad del hueso. El objetivo de este artículo fue describir las nuevas tecnologías en radiología oral para el diagnóstico de la displasia fibrosa monostótica y la importancia de los métodos de imagen actuales para lograr un diagnóstico adecuado. Estas técnicas van desde la radiografía convencional hasta las gammagrafías óseas, que brindan imágenes de mayor calidad, claridad y mejor precisión con técnicas menos invasivas para el paciente. Esta revisión de la literatura ayuda a ampliar el conocimiento de los profesionales de la odontología en relación con las características clínicas y de imagen de la displasia fibrosa monostótica.

## INTRODUCTION

To date no literature review has described the characteristics of monostotic fibrous dysplasia which can be observed by more than three imaging tests. These imaging studies enable adequate diagnosis, with clearer and more precise images, within a shorter time and with less invasive techniques and more optimal storage methods. Fibrous dysplasia is a pseudotumoral lesion that is not related to any complication such as malignant lesions. The affected structures usually only involve bone, and the lesion occurs as a result of the formation of osteofibrous connective tissue or immature fibrous tissue that replaces normal bone marrow.^1^ Fibrous dysplasia can be monostotic when it affects a single bone (higher prevalence), polyostotic when more than one bone is involved, or it can also present as the McCune Albright syndrome^2^ associated with other alterations such as a condition of the endocrine system, light brown pigmented lesions,^3^^,^^4^ and high growth hormone production.^1^

In fibrous dysplasia the facial massif is locally affected and usually presents with swelling, deformity, asymmetry and/or pain. Among the different types of fibrous dysplasia (monostatic fibrous dysplasia, polyostotic fibrous dysplasia and the McCune Albright syndrome), monostotic fibrous dysplasia has the highest incidence regardless of gender or ethnicity and generally affects the maxilla or mandible (molar, premolar and zygomatic process). ^5^

While two-dimensional (2D) images such as panoramic radiographs were already available in the last century, obtaining an adequate diagnosis depends on the technique used and operator knowledge complemented by histopathological examination for differentiation from other bone lesions. Conventional images such as panoramic radiography were initially used to detect monostotic fibrous dysplasia, ^6^ but technological advances now allow more precise diagnosis with the use of cone beam computed tomography. This technique is currently the gold standard diagnostic method because of the advantages it offers with sagittal, coronal and axial slices, including 3D images, showing the characteristic sign of lesions with a ground glass appearance. ^7^

Magnetic resonance imaging is another method able to detect the ground glass and other characteristic signs of fibrous dysplasia. ^8^ Scintigraphy with the venous administration of radiopharmaceuticals and the use of a gamma camera can also determine whether a lesion is isolated or systemic according to the accumulation of the radiotracer in a lesion. ^9^^,^^10^

Monostotic fibrous dysplasia is a rare lesion, and advances in oral radiology techniques, and provide clinical, histopathological and imaging data. These techniques range from conventional radiography to bone scans, which provide images of higher quality, clarity and better precision with less invasive procedures. For these reasons, this literature review will help to expand the knowledge of dental professionals in relation to the clinical and imaging characteristics of monostotic fibrous dysplasia.

## MATERIALS AND METHODS

For bibliographic references, a systematic search was carried out in the main databases of the literature on health sciences, including Medline via PubMed, Scopus, and Lilacs. We reviewed articles published over the period between 2010 and June 2020. The search terms were: monostotic fibrous dysplasia, conventional and digital radiography, cone beam tomography, MRI, and bone scan. We identified important current topics to analyze new concepts which will help dentists and radiologists to know this lesion better.

### The pathophysiology, clinical, imaging and histopathological characteristics of monostotic fibrous dysplasia.

Fibrous dysplasia was first described by Von Recklinghausen in 1891, being further reported by Liechtenstein in 1938 and subsequently described together with Jaffee in 1942 in the article “Fibrous dysplasia of bone: a condition affecting one, several or many bones, the graver cases of which may present abnormal pigmentation of skin, premature sexual development, hyperthyroidism or still other extra skeletal abnormalities” for differentiation from other lesions such as ossifying fibroma and fibrosarcoma, among others.^13^ In advanced states, the disease manifests with pain or increased volume of the affected area and is associated with cafe-au-lait skin spots and precocious puberty.^11^^,^^12^

Monostotic fibrous dysplasia is a congenital disease affecting only a single bone, and disease progression is slow. This type of dysplasia has greater affinity for the upper and lower jaw and usually begins in childhood or puberty affecting the bones and bone marrow. ^10^^,^^14^ The lesion develops when normal bone tissue is replaced by amorphous connective tissue. Abnormal fibroblast production can be observed when porous bone is replaced by another type of bone known as fibrous connective tissue accompanied by bone trabeculae that are very different from those of healthy bone tissue,^15^^,^^16^ leading to disorganization of the organic fibrous bone matrix and causing an alteration in osteogenic cells.^17^ When it is located adjacent to the maxillae or inside the skull, it is called monostotic craniofacial fibrous dysplasia,^10^ and can cause other secondary alterations. For example, on development in the temporal bone, the lesion can manifest with progressive evolution to deafness. ^18^

Histologically, monostotic fibrous dysplasia is observed as a disordered group of trabeculae with indefinite shape resembling Chinese characters with the trabeculae branching out from the fibrous tissue into the medullary spaces and the lamellar structure being accompanied by certain disorganized osteocytes. ^19^

The origin of the development of monostotic fibrous dysplasia has been related to trauma to the affected area or alterations of hormonal origin. It has also been reported to develop as a consequence of alterations in the Gs protein resulting in an increase in cyclic adenosine monophosphate (cAMP) which precludes the formation of osteoblast cells causing disorder of the fibrous bone matrix,^20^ and an increase in the number of mutated cells without the presence of giant multinucleated cells.^21^ Recent studies have reported that it may be due to an alteration of intracytoplasmic protein which alters the gene transducer, and thus, disrupts bone formation within the maternal uterus.^22^

Clinically, monostotic fibrous dysplasia is accompanied by teeth presenting gyroversion, dental crowding and an increase in the dental caries index as a consequence of an alteration at the time of occlusion due to the displacement of neighboring structures.^23^ The patient is usually asymptomatic, and the disease is detected by a gradual increase in volume or enlargement of the area involved, affecting facial esthetics due to asymmetry.^14^ Nonetheless, according to the location and expansion of the disease, in advanced stages, the hearing, sight and adjacent structures may be affected. Some studies have reported alterations in other areas, such as the ethmoid bone (monostotic type), inducing nasal obstruction by deviation of the nasal septum at the level of the middle meatus and part of the middle turbinate. ^24^

Depending on the bone affected and the radiographic findings, 2D images can show a radiopaque image of undefined shape and limits which may be confounded with healthy tissue and do not allow clear visualization of the beginning or end of the lesion. In addition, cortical thinning and expansion (when observed) can displace adjacent anatomical structures which, in advanced cases, can affect normal functioning such as in airway obstruction. ^(14, 20)^

### Useful imaging studies in the diagnosis of monostotic fibrous dysplasia.

With the discovery of X-rays in 1895 radiographic images provided complementary data. Conventional radiographs showed fibrous dysplasia lesions as a change in density with a ground glass appearance. ^25^ The 90s saw the rise of digital radiology, with improvements in the quality, sharpness, and precision of images since then providing greater benefits for patients and health personnel. ^26^

In conventional radiographies, monostotic fibrous dysplasia is shown as mixed or radiopaque images with a blurred appearance which change in density from the outside to the inside or vice versa. These lesions are corroborated by biopsy examination, which can rule out other pathologies such as anosteogenic fibroma. ^25^^,^^27^ Advances in diagnostic methods have evolved from conventional radiographies for detecting monostotic fibrous dysplasia to digital radiographs which are easier to manage and improve the localization of the lesion in 2D images. These images show the lesions as radiopaque with non-precise limits, an absence of defined trabeculae, and demonstrate a possible relationship with adjacent bone structures. ^29^

Another imaging tool is cone beam computed tomography which provides sagittal, coronal and axial three-dimensional (3D) views complemented with 3D bone reconstruction showing the lesion as a hyperdense image (in children) or a mixed mass (in adults) ^7^^,^^14^. The lesion appears as moth-eaten wood, being irregular in shape and with undefined limits. Non-corticalized lesions indicate the direction to which the lesion extends, and the possible displacement of bone structures, cortical erosion or bone reduction. ^30^^,^^31^

Magnetic resonance imaging can be used when a nerve pathway or ligament is affected ^32^ or when the extent of the injury in relation to soft tissues must be determined. According to signal intensity, the lesion may appear hypodense in T1-weighted images and hyperdense in T2-weighted images, with an increase in contrast improving the images in monostotic fibrous dysplasia. In these patients signal intensity in T1-weighted images is intermediate or low (hypodense in isodense lesions), being high (hyperdense) in T2-weighted images. ^33^ The use of magnetic resonance imaging is preferentially used in children due to the absence of radiation. ^(3. 4)^

Finally, bone scintigraphy with a gamma camera and radiotracers can visualize the quality of the existing or absent bone according to the functioning of osteoclastic and osteoblastic cells proportional to the bloodstream in the area.^9^ The presence of a bone fracture can also be observed if the injury is related to bone structures, and bone scintigraphy can determine lesion extension and activity.^35^ In bone scintigraphy radioactive material is intravenously injected (after allergy testing) and the gamma rays given off by the radiotracer are detected by the gamma camera, demonstrating increased or decreased uptake in the lesion or adjacent tissues.^36^ The images are obtained at 3 time points: at the time of injection, after a few minutes, and finally, after 3 to 5 hours, pinpointing uptake of the radiotracer within the lesion and indicating possible extension in the earliest stages of the disease.^37^

## Differential diagnoses of monostotic fibrous dysplasia and the importance of imaging in the process.

Monostotic fibrous dysplasia is a non-malignant lesion that is characterized by imaging studies as a radiopaque image indicating a hyperdense mass that is common in bone lesions, making differential diagnosis with other lesions with similar characteristics essential.

Ossifying fibromas are benign bone lesions located only in the skull and should be differentiated from fibrous dysplasia. Similar to monostotic fibrous dysplasia, these lesions are constituted by the formation of fibrous tissue cells which are slow-growing, asymptomatic and benign. Nonetheless, ossifying fibromas are radiologically considered to be tumors ^38^, showing radiolucent, mixed or radiopaque images with defined limits and sclerotic borders. The difference between ossifying fibromas and monostotic fibrous dysplasia is that the borders of the latter are diffuse and non-corticalized. In addition, tomographic sections show more defined images with the presence of cortical and bone trabeculae that varies according to the evolution of the lesion and conditions the displacement of neighboring structures. ^21^^,^^39^

Cherubism is an asymptomatic and painless hereditary lesion which presents an increase in volume similar to what occurs in monostotic fibrous dysplasia. However, panoramic radiography and cone beam computed tomography ^21^ show numerous cysts that mainly affect the jaw. Cherubism is an autosomal dominant disease involving an alteration of the *SH3BP2* gene related to the regulation of osteoclasts and osteoblasts. ^40^

In addition, cement-ossifying fibroma is an uncommon benign odontogenic disease. Radiological images show defined borders, which according to the stage of its evolution are multilocular in early stages and may be found as unilocular radiolucent images over time. Cone beam computed tomography shows different levels of radiopacity, which, in general, do not displace any type of hard tissue or erode adjacent structures. ^41^

In the differential diagnosis of monostotic fibrous dysplasia, florid cement-osseous dysplasia, or florid osseous dysplasia is also a benign disease which develops in the maxilla or the mandible. Both lesions can be observed in panoramic radiography as a radiopaque, radiolucent, or mixed image. Florid osseous dysplasia presents a radiolucent halo and is predisposed to the development of infection. In addition, its presentation is multi-quadrant, unlike monostotic fibrous dysplasia, which presents in a single quadrant. ^42^

Paget's disease is an asymptomatic condition which can present with pain of muscular, neurological or bone origin, associated with endocrinological alterations such as a deficit of vitamin D. This disease is rare in children, being more prevalent in adults. It involves cellular remodeling and deformity of bone structures such as the mandible or maxilla, inducing poor bone quality prone to fractures. ^43^ In early stages of the disease, panoramic radiography shows multifocal mixed images which evolve and change in appearance to become fluffy or cotton-like and irregular in shape. Diffuse, non-corticalized boundaries are observed differing from monostotic fibrous dysplasia, which presents as a single lesion. In cone beam computed tomography, Paget's disease can show expansion of the lingual and vestibular tables, accompanied by hypercementosis of the teeth, and in severe cases, areas with resorption with possible progression to osteomyelitis. ^44^ As in monostotic fibrous dysplasia, bone scintigraphy in Paget’s disease shows greater radiotracer uptake (20 mCi of 99mTc-methylene diphosphonate^)^ only in the region of the lesion. ^43^

Renal osteodystrophy directly affects the kidneys but is accompanied by certain manifestations in bones such as the maxilla or mandible, with a delay in the eruption of the permanent teeth. Similar to monostotic fibrous dysplasia, panoramic radiographies in renal osteodystrophy show ground glass or radiopaque lesions which are predisposed to fracture. Resorption of the lamina dura and tooth mobility are also observed. ^43^

## DISCUSSION

Monostotic fibrous dysplasia is located in bone structures and, being an asymptomatic disease, it is detected in advanced stages, thereby making it important to know the characteristics of the evolution of the disease and how it is observed in different diagnostic imaging methods in order to select the most adequate study to provide the greatest benefits.

Monostotic fibrous dysplasia is a lesion with benign characteristics that affects hard tissue (bone) but can compromise neighboring structures depending on the time of evolution in which it is detected and according to its location ^10^^,^^13^.

A disadvantage of 2D images is the superposition of structures, although this does prevent the detection of asymptomatic lesions. Likewise, while magnetic resonance imaging does not emit radiation, the efficiency of this technique is limited to only the compromised soft tissue since the identification of bone tissue lesions with this method remains to be described ^33^. Bone scintigraphy is limited to determining whether the lesion occurs in any other bone or has spread to other areas. Thus, cone beam computed tomography continues to be the most effective diagnostic method because of its utility in evaluating the lesion and establishing its relationship with adjacent structures. ^9^^,^^25^^,^^27^

According to the literature, different pathologies can be differentiated from monostotic fibrous dysplasia with the use of sequential or selective imaging methods. Nonetheless, the use of panoramic radiography combined with computed tomography provides greater information in relation to the description of the characteristics of this disease. ^40^^,^^41^^,^^42^

Although there were difficulties in collecting information on the exclusive imaging characteristics of the disease, articles related to other differentiated pathologies were reviewed and more specific literature was included for study.

It is recommended to continue updating the description of monostotic fibrous dysplasia with different imaging diagnostic methods that involve less radiation and less waiting time and greater precision in order to detect this disease earlier and successfully differentiate it from other diseases.

## CONCLUSION

Monostotic fibrous dysplasia is a benign pathology with well-known clinical and imaging characteristics, and technological advances have led to a reduction in the differential diagnoses for this disease. Diagnosis can be achieved with different imaging methods such as conventional radiography, digital radiography, cone beam computed tomography, magnetic resonance and bone scintigraphy. However, cone beam computed tomography is currently the method of choice due to its lower cost, accuracy and accessibility, although magnetic resonance can provide complementary information for obtaining a more accurate diagnosis. Nonetheless, definitive diagnosis of monostotic fibrous dysplasia is obtained by histopathological examination.
